# Whole exome sequencing in adult-onset hearing loss reveals a high load of predicted pathogenic variants in known deafness-associated genes and identifies new candidate genes

**DOI:** 10.1186/s12920-018-0395-1

**Published:** 2018-09-04

**Authors:** Morag A. Lewis, Lisa S. Nolan, Barbara A. Cadge, Lois J. Matthews, Bradley A. Schulte, Judy R. Dubno, Karen P. Steel, Sally J. Dawson

**Affiliations:** 1 Wolfson Centre for Age-Related Diseases, King’s College London, WC2R 2LS, London, UK; 2 Wellcome Trust Sanger Institute, Hinxton, Cambridge, CB10 1SA UK; 3 UCL Ear Institute, University College London, WC1X 8EE, London, UK; 4 Medical University of South Carolina, Charleston, SC 29425 USA

**Keywords:** Hearing loss, Whole exome sequencing, Deafness

## Abstract

**Background:**

Deafness is a highly heterogenous disorder with over 100 genes known to underlie human non-syndromic hearing impairment. However, many more remain undiscovered, particularly those involved in the most common form of deafness: adult-onset progressive hearing loss. Despite several genome-wide association studies of adult hearing status, it remains unclear whether the genetic architecture of this common sensory loss consists of multiple rare variants each with large effect size or many common susceptibility variants each with small to medium effects. As next generation sequencing is now being utilised in clinical diagnosis, our aim was to explore the viability of diagnosing the genetic cause of hearing loss using whole exome sequencing in individual subjects as in a clinical setting.

**Methods:**

We performed exome sequencing of thirty patients selected for distinct phenotypic sub-types from well-characterised cohorts of 1479 people with adult-onset hearing loss.

**Results:**

Every individual carried predicted pathogenic variants in at least ten deafness-associated genes; similar findings were obtained from an analysis of the 1000 Genomes Project data unselected for hearing status. We have identified putative causal variants in known deafness genes and several novel candidate genes, including *NEDD4* and *NEFH* that were mutated in multiple individuals.

**Conclusions:**

The high frequency of predicted-pathogenic variants detected in known deafness-associated genes was unexpected and has significant implications for current diagnostic sequencing in deafness. Our findings suggest that in a clinic setting, efforts should be made to a) confirm key sequence results by Sanger sequencing, b) assess segregations of variants and phenotypes within the family if at all possible, and c) use caution in applying current pathogenicity prediction algorithms for diagnostic purposes. We conclude that there may be a high number of pathogenic variants affecting hearing in the ageing population, including many in known deafness-associated genes. Our findings of frequent predicted-pathogenic variants in both our hearing-impaired sample and in the larger 1000 Genomes Project sample unselected for auditory function suggests that the reference population for interpreting variants for this very common disorder should be a population of people with good hearing for their age rather than an unselected population.

**Electronic supplementary material:**

The online version of this article (10.1186/s12920-018-0395-1) contains supplementary material, which is available to authorized users.

## Background

Hearing loss is one of the most common sensory deficits in the human population, and it has a strong genetic component [1]. However, although more than 140 human non-syndromic hearing impairment loci have been mapped and over 100 genes identified, most underlie childhood deafness or early-onset hearing loss. The vast majority of genes involved in hearing remain unknown, including those associated with adult-onset, age-related progressive hearing loss. Age-related hearing loss (ARHL) affects 1 in 3 people over the age of sixty, often leading to social isolation and depression, is associated with subsequent cognitive decline [2–4] and a predictor of dementia [5]. The heritability of ARHL has been estimated to be between 30 and 50%, similar to other common complex disorders [1, 6, 7].

Although several ARHL genome-wide association studies (GWAS) have been carried out [8–11] and promising candidate genes identified, such as *SIK3* and *ESRRG* [10, 11], only five loci have been associated with hearing status at the genome-wide significance level: *GRM7* [12], *PCDH20* and *SLC28A3* [13], and *ISG20* or *ACAN* and *TRIOBP* [14]. Furthermore, single genes can underlie progressive hearing loss with post-lingual onset, including in middle-age, particularly genes underlying dominantly-inherited deafness [15, 16]. Thus, adult-onset hearing loss may result from either rare Mendelian gene variants with large effect size or multiple variants each making a small contribution to hearing loss. It is also unclear whether these variants are in novel genes involved in maintenance of auditory pathways or whether they are milder variants of the same genes that are mutated in congenital deafness.

Here, we have taken a more in-depth approach than GWAS, using whole-exome sequencing (WES) to study thirty patients carefully selected from a total sample of 1479 patients with a variety of adult-onset hearing loss phenotypes to represent the mixed phenotypes and varied genetic aetiology that might be present in a clinical scenario, targeting specific sub-phenotypes to maximise power to detect shared variants. Our aim was to establish to what extent exome sequencing is an effective and appropriate tool for genetic diagnosis of hearing loss in a clinic setting, where there is usually only a single adult patient involved and family members are not available for segregation analysis. Whole exome and genome sequencing are beginning to be used in this scenario for diagnosis of adult-onset hearing loss with the clinician faced with challenges in evaluating the candidate variants identified. Our results demonstrate the value of targeting well-characterised phenotypic subtypes and cross-species data comparison in exome sequencing analysis, and highlight issues which need to be considered in interpreting genetic variants of unknown pathogenicity in current genetic diagnosis and gene discovery studies, in particular the finding that many individuals have multiple predicted-pathogenic variants in different genes known to underlie deafness.

## Methods

### Recruitment of patients

Twenty patients (seven males, thirteen females) with non-syndromic sensorineural adult-onset hearing loss (self-reported age of onset between 20 and 50) were selected from a larger group of 700 patients recruited from the adult hearing aid clinic at the Royal National Throat Nose and Ear Hospital, London, U.K. (described in [17]). The twenty were chosen based on a family history of hearing loss and an age of onset in middle age. Air and bone conduction thresholds at 0.25, 0.5, 1, 2, 4 and 8 kHz and 0.5, 1, 2 and 4 kHz, respectively were measured with masking as indicated according to BSA Recommended Procedures [18] (Additional file 1: Figs. S1, and Additional file 2: Figs. S2). Ethical approval for this project was granted from the Royal Free Local Research Ethics Committee (reference 6202).

A second group of 10 older individuals were selected from the 779 people in the database of the longitudinal study of ARHL being conducted at the Medical University of South Carolina (MUSC) since 1987 (described in [19]). These individuals were selected for the current analyses on the basis of age (> 60 and < 79 years), negative or limited self-reported occupational and recreational noise history, available DNA samples, and audiometric phenotype (five metabolic: 4 females, 1 male; mean age 69.8 years and five sensory: 2 females, 3 males; mean age 68.3 years) (Additional file 3: Fig. S3). The protocols for this study were approved by the Institutional Review Board at MUSC.

All patients were recruited by written informed consent.

### Exome sequencing

DNA was submitted for WES using either the Agilent SureSelect Human All Exon V3 kit or a custom library designed by Agilent for human whole exome sequence capture (which predates the SureSelect kit). Sequencing was carried out on either the Illumina Genome Analyzer IIx or Illumina HiSeq 2000 platform as paired-end 54 bp, 75 bp or 76 bp reads according to the manufacturer’s protocol.

**Fig. 1:**
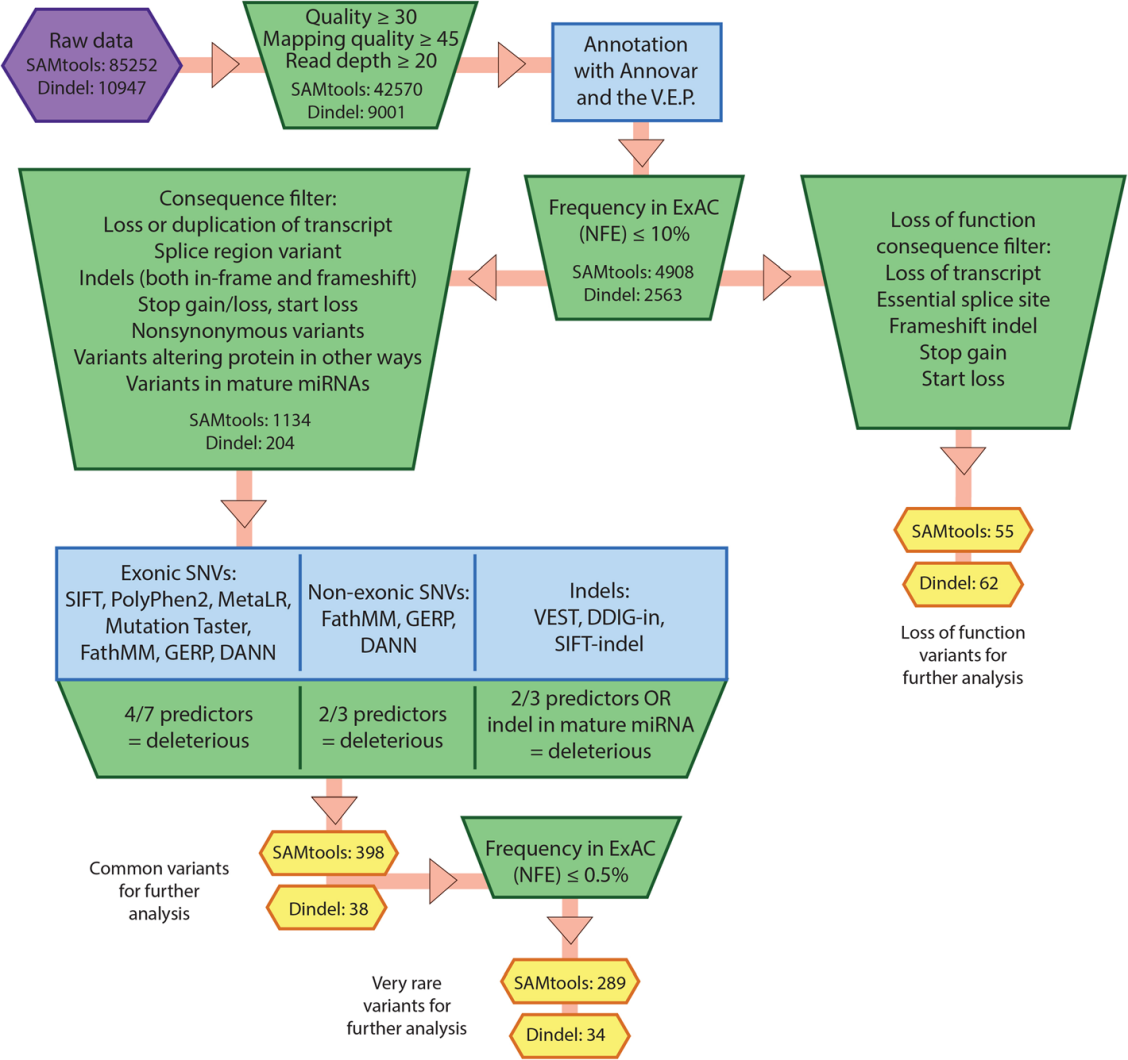
Filtering pipeline. Diagram showing the filtering pipeline used for processing and analysing data. Mean variant counts are shown at each stage

The data were filtered on quality, Minor Allele Frequency (MAF) in the ExAC non-Finnish European population [20], most severe consequence and predicted pathogenicity using the pipeline shown in Fig. 1 (Fig. 1, Additional file 4: Table S1). Where no data were recorded in ExAC but a MAF was available from the 1000 Genomes European population [21], e.g. for rs1813100, this was used. The impact of filtering on variant number is shown in Additional file 5: Fig. S4. Following filtering, variants were examined both individually and by gene (using only genes and known miRNAs present in Ensembl, accessed May 2016), and the gene lists analysed to find those mutated genes shared between individuals.

### Variant confirmation

Candidate variants were re-sequenced using Sanger sequencing (Source Bioscience). Primers were designed using Primer3 [22] (Additional file 6: Table S2). Sequence data were analysed using Gap4 [23].

### 1000 Genomes project data

Genotype and annotation data from the 1000 Genomes project [21, 24] were used to create files of gene variants from each of the 2504 individuals sequenced in the project. Variants were processed using the same pipeline (Fig. 1), with the exception of the quality filter (quality of variant call ≥30, read depth ≥ 20, mapping quality ≥45), which was omitted because these variant calls have already been validated and filtered [21].

## Results and discussion

In order to determine the possibility of using WES to reveal the genetic basis of adult onset hearing loss in a typical clinical scenario, we identified 30 individuals with different phenotypic sub-types based on family history, age of onset and audiogram shape from large well characterised cohorts. Twenty patients with family histories were subdivided into ten with probable dominant hearing loss and ten with presumed recessive hearing loss. Ten older adults without a family history of hearing loss were subdivided into five people with a metabolic phenotype of ARHL and five with a sensory phenotype of ARHL based on audiogram shape [19]. These sub-groups are henceforth referred to as *Dominant, Recessive, Metabolic* and *Sensory* respectively.

Because hearing loss is common, we selected a low-stringency allele frequency filter of 10%, to detect common risk variants in our phenotypic sub-groups. We compiled a list of known deafness genes comprising all genes listed in the Hereditary Hearing Loss Homepage [15] plus the human orthologues of all the mouse deafness genes from the Hereditary Hearing Impairment in Mice website (described in [25]); 357 genes in total (Additional file 7: Table S3). Mutant mice continue to be valuable tools for discovering genes required for hearing [25], many of which have subsequently been shown to also underlie deafness in humans, such as *WBP2* [26] and *MIR96* [27, 28].

To identify potential false positives due to platform errors we utilised a list of 507 such genes described for the Illumina Genome Analyser IIx by Fuentes Fajardo et al. [29] (Additional file 8: Table S4). We have not excluded these genes from our analysis but have marked them as “candidates for exclusion” where they occur.

### First analysis: Common variants

Here, common variants that were predicted to be pathogenic (≤10% MAF in the non-Finnish European population) were analysed to find genes common within each sub-group.

### Known deafness genes in all four sub-groups combined

**Fig. 2:**
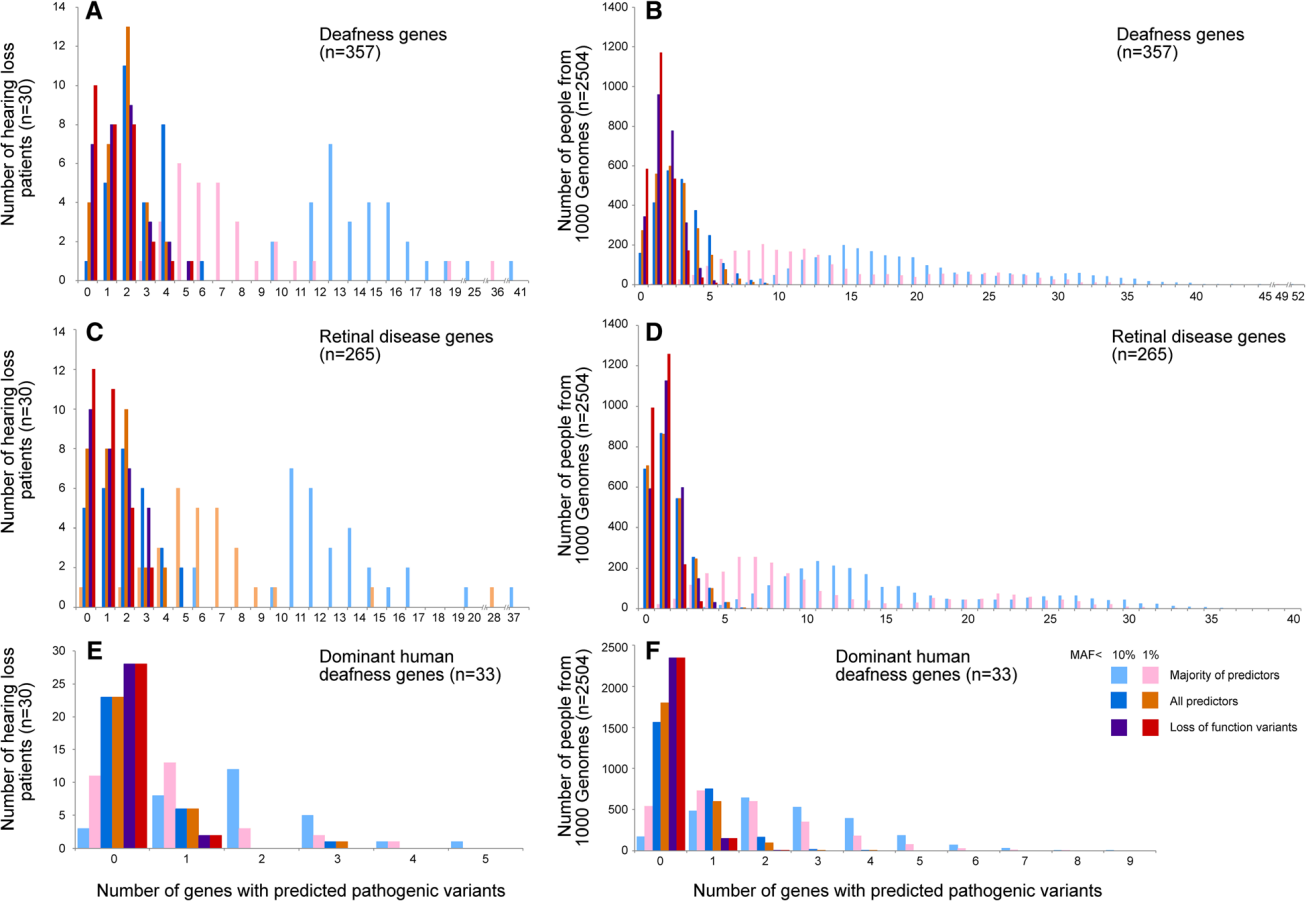
Numbers of individuals with predicted pathogenic mutations in deafness and retinal disease genes. Numbers of deafness (**a, b**), known dominant deafness (**e, f**) and retinal disease (**c, d**) genes bearing predicted pathogenic variants in thirty patients with hearing loss (**a, c, e**) and in the 2504 individuals sequenced in the 1000 Genomes study (**b, d, f**). Variations with a Minor Allele Frequency of 10% or lower in the non-Finnish European population are shown in purple, dark blue, pale blue, and those with a MAF of 1% or lower in the non-Finnish European population in pink, orange and red; pale blue and pink indicate the variants were predicted pathogenic by a majority of predictors, dark blue and orange indicate the variants were predicted pathogenic by all predictors, and red and purple indicate the variants are classified as loss of function variants

We first examined known deafness genes in all thirty people together and found that every person had at least ten known deafness genes with one or more predicted pathogenic variants (Fig. 2A, pale blue bars). The same analysis on data from the 1000 Genomes project, which includes exome sequences from 2504 people with unknown auditory function, produced a very similar distribution of predicted pathogenic variants to the 30 people with adult-onset hearing loss (Fig. 2B). As this high frequency of predicted pathogenic variants was an unexpected finding, we asked if the same pattern of distribution was present in genes known to be involved in retinal disease, another sensory deficit with a large number of single genes known to be involved. We repeated the analysis using 265 retinal disease genes (from RetNet [30, 31], Additional file 9: Table S5) instead of our list of deafness genes. Again, we found a very similar distribution of predicted pathogenic variants in both our thirty patients with hearing loss and in the 2504 samples from the 1000 Genomes project (Fig. 2C, D). We then looked at human dominant deafness genes only (*n* = 33, Additional file 7: Table S3), where pathogenic variants would be expected to have an effect even when only present in one allele. We found once again a similar distribution of variants in our thirty patients and the 1000 Genomes samples (Fig. 2E, F) and 27 of our 30 patients had at least one predicted pathogenic variant in a dominant deafness gene (Fig. 2C).

To test whether this finding was due to having too many common variants, we repeated the analysis using a 1% MAF cut-off (instead of 10%), and found the same pattern (Fig. 2, pink bars). We then tested a more stringent filter for pathogenicity, selecting only those variants predicted to be pathogenic by *all* the predictors used instead of the majority of predictors. The distributions were again similar for all groups of people and sets of disease-associated genes with both MAF filters (Fig. 2, dark blue and orange bars for MAF < 10% and MAF < 1% respectively). Finally, we looked for only loss of function variants (loss of transcript, essential splice site, frameshift indel, stop gain or start loss, see Fig. 1), and again observed a similar distribution (Fig. 2, purple and red bars for MAF < 10% and MAF < 1% respectively). Two of our patients and 152 people from the 1000 Genomes project had a loss of function variant in a known human dominant deafness gene (Fig. 2E, F). The two patients were both from the dominant sub-group; one had a single base pair insertion (causing a frameshift) in *MYO6* and the other had a nonsense mutation in *HOMER2*.

### Genes common to individual sub-groups

We then looked at individual genes with predicted pathogenic variants (MAF < 10%, predicted by a majority of predictors) present in all members of each sub-group. No known deafness genes were common to any of the sub-groups. We found four other such genes: *TTN* (a candidate for exclusion) bore variants in all ten individuals with recessive hearing loss, *MON1B* was mutated in all five individuals with metabolic deafness (but see later), and the five people with sensory hearing loss all had variants in *NEDD4* and *ZAN* (Table 1).

**Table 1:** Genes with common variants (MAF < 10%) in all the members of a subgroup

Gene	Sub-group	Number of variants	Type of variants
*TTN*	Recessive	22	Non-synonymous
*MON1B*	Metabolic	1	Frameshift
*NEDD4*	Sensory	2	Non-synonymous
*ZAN*	Sensory	7	Six non-synonymous, one in-frame deletion

Details of the four genes found to be mutated in all members of a subgroup. *TTN* (underlined) is a candidate for exclusion

### Implications for WES analyses in common disease

Our common variant analysis has highlighted several potential problems with detecting common variants of small effect size in a common disorder. Although each individual had many deafness genes with predicted pathogenic variants, the overall spread didn’t look any different to that observed in the 1000 Genomes dataset [21] (Fig. 2). However, the 1000 Genomes data do not exclude people with hearing loss, particularly adult-onset hearing loss which may not be evident at the time of sampling an individual. This highlights the need for good controls in this type of analysis, in both clinics and research; suitable controls in this case might be older adults with good hearing typical of a younger adult. Other exome sequencing projects have reported similar results for age-related macular degeneration [32] and in the ExAC data an abundance of rare, functional variants were reported in many disease genes [20]. It has been hypothesised that both false pathogenicity reports and incomplete penetrance contribute to this over-reporting, but whatever the reason, putative causative variants must be treated with caution until proof of pathogenicity has been obtained, preferably by functional studies and linkage analysis.

Another factor to consider is whether variants which are rare in the most relevant ethnic populations and passed the MAF filter but are more prevalent in other populations should be retained; 1234 of the 10,482 unique variants in the thirty patients which passed all our filtering steps were present in other ExAC populations at a MAF of more than 10%. To give one extreme example, it is worth considering whether a variant which is present in 60% of the African ExAC population should be included even if it is very rare (0.3%) in the non-Finnish European population, as for a T > C missense variant in *LPP* (chr3:g.188327555 T > C).

### Second analysis: Very rare variants

Since the pattern of common variants in our thirty patients with hearing loss did not differ from that observed in the data from the 1000 Genomes project, we then filtered for very rare variants with a minor allele frequency of < 0.5%, predicted to be deleterious to protein function by a majority of predictors to identify any likely causal variants. This is the recommended MAF filter for recessive hearing loss (the recommended MAF for dominant hearing loss is 0.05%) [33]. No individuals from any of the groups, including those with a strong family history consistent with recessive inheritance, were homozygous for a variant in a known deafness gene. Apparently-homozygous variants were found in 29 other genes in only one of the individuals, and two genes bore homozygous variants in multiple people: *SIRPA* (a candidate for exclusion) and *ZAN* (Additional file 10: Table S6).

**Fig. 3:**
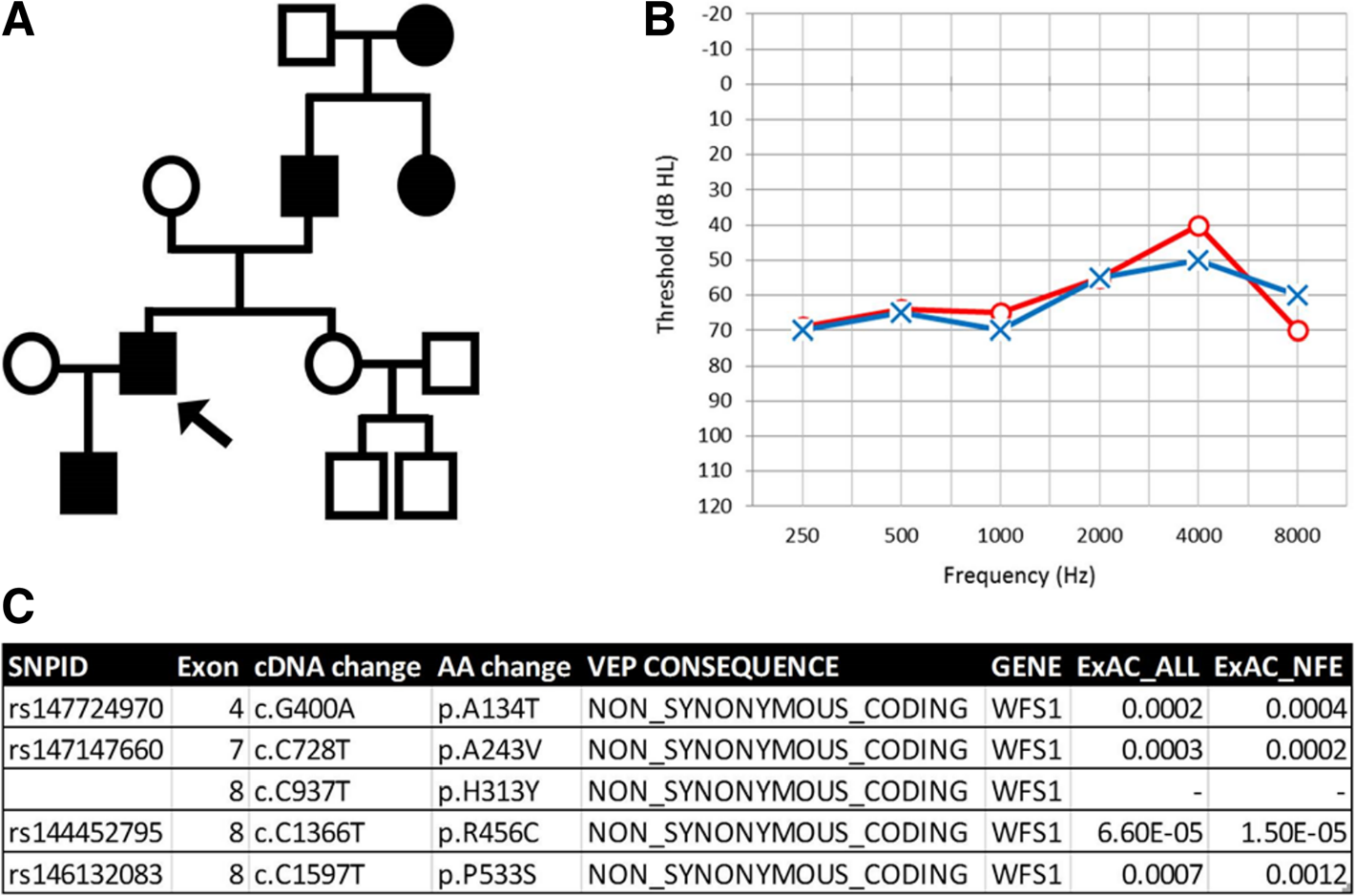
*WFS1* mutations in a single patient. Patient 2590 has 5 missense mutations in the *WFS1* gene. **a** shows a pedigree of patient 2590 (who is designated by an arrow), showing the hearing loss inherited in a pattern consistent with a dominant mechanism. **b** shows the audiogram of individual 2590 at age 62, crosses and circles show the thresholds of the left and right ear, respectively. **c** describes all 5 mutations in *WFS1* and the predicted effect on Ensembl transcript ID ENST00000226760. The mutation predicted to cause a p.H313Y change has been described before in patients with Wolfram Syndrome

Many known deafness genes were mutated in multiple samples in the heterozygous state (Table 2); for example, 7 individuals bore variants in *GPR98*. One individual from the dominant group had five predicted variants in *WFS1* (Fig. 3), and many people bore more than one variant in the same gene, including three individuals from the recessive group who bore two heterozygous variants in a deafness gene (Additional file 11: Table S7). These variants might explain the hearing loss seen in these three patients, but with one exception, without segregation analysis it isn’t possible to tell whether the variants are on the same chromosome or were inherited one from each parent. The exception is the two variants in *GPR98* in sample 11,813, which are close enough to fall within the same sequencing read, and can be confirmed to originate from the same chromosome. Furthermore, several very rare variants were also found to be present in more than one individual (Additional file 12: Tables S8 and Additional file 13: Table S9).

**Table 2:** Genes with very rare predicted pathogenic variants in more than one person

Number of individuals	Genes					
12	*MON1B*					
11	*TTN*					
8	*ADC*	*NEFH*	*ZAN*			
7	*DNAH2*	***GPR98***	*LRBA*	***PAX2***		
6	*CHD3*	*DNAH3*	*MACF1*	*PTGER4*	*UBE2O*	*NEB*
	*WDR19*	*ZMIZ2*				
5	*VWA5B1*	*DNAH8*	*HSPG2*	*DNAH1*	*CELSR3*	*PCNX*
	*DNAH7*	*PKHD1L1*	***TECTA***	*ATG2A*	*ATM*	*RANBP17*
	***LRIG3***	*DNAH9*	*HECTD4*	*OBSCN*	*VPS13B*	*FAT2*
	***LAMA2***	*CAPN5*	***CDH23***	*WDR41*		
4	***DMD***	***DUOX2***	***RBPJ***	***USH2A***	***MYO6***	*+52* ^*a*^
3	***COL11A1***	***NAV2***	***CPXM2***	***COL4A4***	***LRP2***	***MY015A***
	***MYH9***	***TSPEAR***	***ACAN***	***PCDH15***	***OTOG***	*+160* ^*a*^
2	***WFS1***	***MECOM***	***NTN1***	***GJB2***	***TCOF1***	***COL11A2***
	***CELSR1***	***SLC9A3R1***	***COL9A1***	***TJP2***	***ALMS1***	***JAG1***
	***ATP2B2***	***SLC26A4***	***LRIG1***	***LOXHD1***	***CHRNA9***	***RDX***
	***CHD7***	***NTF3***	***ELMOD3***	***SLC4A7***	***ATP8B1***	***NPC1***
	***KARS***	***ERCC6***	*+745* ^*a*^			

Details of genes found to be mutated in multiple samples. Known deafness genes are in bold, and candidates for exclusion are underlined.

^a^Number of additional non-deafness genes with variants; only known deafness genes shown for these lists

When we examined heterozygous variants in all genes, we found several genes bearing rare variants in six or more people (Additional file 13: Table S9). One of particular interest was *NEFH*, which encodes the neurofilament heavy chain and is strongly expressed in the spiral ganglion neurons innervating the inner hair cells [34].

We also examined the eight strongest candidate genes (*PCDH20, GRM7, ESRRG, SIK3, SLC28A3*, *TRIOBP*, *ISG20* and *ACAN*) linked to adult hearing ability or ARHL in GWAS [10–14]. Five rare variants were found: a novel deletion predicted to cause a frameshift in *GRM7*, a missense variant in *SIK3*, and three missense variants in *ACAN*, each present in one individual except for one of the variants in *ACAN*, which is present in two people (Additional file 14: Table S10).

### Resequencing

We chose 29 variants for confirmation by Sanger sequencing, covering fourteen genes, for our final quality control step. We focussed on variants in the genes common to phenotype-specific subgroups (*NEDD4*, *MON1B*, *ZAN*, Table 1), genes with rare variants in multiple people (*SIRPA*, *ZAN*, *MON1B*, *NEFH*, *ADC*, *GPR98*, *LRBA*, *PAX2* and *DNAH2*, Table 2 and Additional file 10: Table S6), genes with identical variants in multiple people (*PAX2*, *RBPJ*, *MON1B*, *NEFH*, Additional file 12: Tables S8 and Additional file 13: Table S9), variants in GWAS genes (*GRM7*, *SIK3*, Additional file 14: Table S10) and *WFS1*, which had 5 variants in one individual (Additional file 11: Table S7). Where genes had multiple predicted variants, we focussed on those present in more than one individual.

Approximately two thirds of our selected variants were confirmed (Table 3 and Additional file 15: Table S11), although the predicted zygosity was not always correct. Most failures were single base pair indels called by Dindel, but three were SNVs called by SAMtools (Table 3). Four additional indels called by Dindel were present but found to be different to the Dindel prediction. For example, eight patients were predicted to bear a single base pair insertion in *NEFH*, which would result in a frameshift, but in fact Sanger sequencing detected an 18 bp insertion, which is not a frameshift. This miscalling also leads to incorrect minor allele frequencies being associated with each variant (compare the predicted MAF with the MAF of the confirmed variants in Table 3). None of the confirmed indels were detected by SAMtools, even though it is capable of calling small indels. Our findings suggest that while it is valuable to include Dindel, its output should be used with care in variant calling pipelines. In summary, we found a surprisingly high level of false calls from the exome sequencing, confirming that Sanger sequencing should always be used to verify important variants.

**Table 3:** Results of resequencing chosen variants

Gene	Caller	WES Predicted variant	Predicted	Confirmed variant	Patients with variant		Variant ID	MAF	Consequence
			MAF		Heterozygote	Homozygote			
NEDD4	Samtools	g.15:56208463A > C	0.0356	g.15:56208463A > C	1D 3S		rs113176671	0.038	Missense
NEDD4	Samtools	g.15:56208933 T > C	0.0992	g.15:56208933 T > C	3D 1S		rs1912403	0.108	Missense
ZAN	Dindel	g.7:100385563delC	–	g.7:100385563_100385597del		3D	rs369526619	0.456^a^	Frameshift deletion
ZAN	Samtools	g.7:100371417G > A	0.0451	g.7:100371417G > A	1 M 1R		rs12673041	0.032	Missense
ZAN	Dindel	g.7:100353014_100353016del	0.0548	g.7:100353019_100353021del	2R		rs377643276	–	Inframe deletion
ZAN	Samtools	g.7:100346094G > A	0.0476	g.7:100346094G > A	1D		rs117406702	0.032	Essential splice site
ZAN	Samtools	g.7:100389590C > T	0.0803	g.7:100389590C > T	1S		rs76325149	0.085	Missense, splice site
DNAH2	Samtools	g.17:7736480 T > A	0.0095	g.17:7736480 T > A	1D 1R		rs78354379	0.008	Missense
GPR98	Samtools	g.5:89925039A > C	0.0035	g.5:89925039A > C	2D		rs61744480	0.002	Missense
GPR98	Samtools	g.5:90106415 T > G	0.000015	g.5:90106415 T > G	1R		–	–	Missense
NEFH	Dindel	g.22:29885564_29885564insG	–	g.22:29885568_29885585dup	1R 1S	3D 2R 1S	rs147489453	0.577	Inframe duplication
NEFH	Samtools	g.22:29885016G > A	0.0971	g.22:29885016G > A	2D 1R	1D	rs59371099	0.1	Missense
WFS1	Samtools	g.4:6290798G > A	0.0004	g.4:6290798G > A	1D		rs147724970	0.0002	Missense
WFS1	Samtools	g.4:6296783C > T	0.0002	g.4:6296783C > T	1D		rs147147660	0.00013	Missense
WFS1	Samtools	g.4:6302459C > T	–	g.4:6302459C > T	1D		rs886044563	–	Missense
WFS1	Samtools	g.4:6302888C > T	0.000015	g.4:6302888C > T	1D		–	–	Missense
WFS1	Samtools	g.4:6303119C > T	0.0012	g.4:6303119C > T	1D		rs146132083	0.001	Missense
GRM7	Dindel	g.3:7494306delT	–	g.3:7494272_7494273del	1 M		–	–	Frameshift deletion
SIK3	Samtools	g.11:116728913G > A	0.0002	g.11:116728913G > A	1 M		rs61738656	0.0002	Missense
MON1B	Dindel	g.16:77228709_77228710insG	–	Failed	n/a	n/a			
ADC	Dindel	g.1:33583668_33583669insG	0.0005	Failed	n/a	n/a			
ADC	Dindel	g.1:33583674_33583675insC	3.24E-05	Failed	n/a	n/a			
PAX2	Dindel	g.10:102587323dupC	4.58E-05	Failed	n/a	n/a			
PAX2	Dindel	g.10:102587329dupG	–	Failed	n/a	n/a			
PAX2	Dindel	g.10:102587333dupC	–	Failed	n/a	n/a			
SIRPA	Dindel	g.20:1895964_1895965del	–	Failed	n/a	n/a			
LRBA	Samtools	g.4:151727540C > A	0.0028	Failed	n/a	n/a			
LRBA	Samtools	g.4:151837565G > T	0.0011	Failed	n/a	n/a			
RBPJ	Samtools	g.4:26426018C > G	0	Failed	n/a	n/a			

Details of variant validation. The number of heterozygous and homozygous patients with each variation is annotated with the subgroups to which each belonged, where “D” stands for the 10 patients with dominantly inherited hearing loss, “R” for the 10 patients with recessive hearing loss, “M” for the 5 patients with a metabolic phenotype and “S” for the 5 patients with a sensory phenotype. Predicted variant MAF is from the 1000 Genomes data [24]; confirmed variant MAF is from gnomAD genome data [20], except for ^a^, which is from the NHLBI GO Exome Sequencing Project [70]

### Candidate genes

Eight genes had confirmed variants, including four candidate novel deafness genes; *NEDD4*, *ZAN*, *DNAH2*, and *NEFH*. The four known deafness genes with confirmed variants are *GPR98*, *WFS1*, *GRM7* and *SIK3*.

Details of the 9 predicted variants in *GPR98* are described in Additional file 16: Table S12, including those found in multiple individuals (Table 2), as well as 2 additional variants found in individuals who carry multiple variants in this gene (Additional file 11: Table S7). *GPR98* is a large gene (90 exons encoding 6307 amino acids) encoding a G protein coupled receptor, and frameshift mutations in this gene cause one form of Usher’s syndrome, USH2C, an autosomal recessive disorder [35] causing congenital hearing impairment and retinitis pigmentosa (OMIM #605472). The variants described here in patients without retinitis pigmentosa are heterozygous missense variants, plus one in a splice site (Additional file 14: Table S10), and these are spread throughout the protein from exons 9 to 89. The two missense SNVs we sequenced were confirmed in the three patients predicted to carry them.

All five predicted variants in *WFS1* in individual 2590 were confirmed by resequencing. Mutations in *WFS1* can cause either a dominantly-inherited non-syndromic hearing loss typically affecting the low frequencies (below 2 kHz) or Wolfram Syndrome (OMIM #222300), a recessive neurodegenerative disease which can include mental retardation, childhood diabetes, optic atrophy and deafness which typically is progressive and affects the high frequencies [36]. The family history, medical history and audiogram shape of individual 2590 are consistent with a dominant non-syndromic hearing loss rather than Wolfram Syndrome (Fig. 3). All five mutations are predicted to be pathogenic but only one of the mutations (p.His313Tyr) has been reported previously, in the heterozygous state in 4 families with Wolfram Syndrome [37, 38]. Individuals previously reported with this variant are all deaf, some also had mental retardation but not all, this mutation is reported as a probably pathogenic mutation in the Wolfram Syndrome Mutation Database (https://lovd.euro-wabb.org/home.php?select_db=WFS1).

Both variants in two of the genes linked to hearing loss by previous GWAS reports were confirmed, in two patients from the metabolic subgroup, one with a deletion in *GRM7* and one with a *SIK3* SNV (Table 3). *GRM7* codes for a metabotropic glutamate receptor, and is expressed in the spiral ganglion and the cochlear and vestibular hair cells in the mouse [12]. The variant was predicted to be a deletion of a T, but we found instead a deletion of TG 34 bp 5′ of the prediction and 22 bp 5′ of the acceptor splice site for exon 6 (transcript ENST00000357716), which makes it an intronic variant and unlikely to affect protein function. *SIK3* is a salt-inducible kinase, and is also expressed in the mouse spiral ganglion and cochlear hair cells, although only in young mice [11]. The contribution of these heterozygous variants to the hearing loss of the patients bearing them is unclear.

**Fig. 4:**
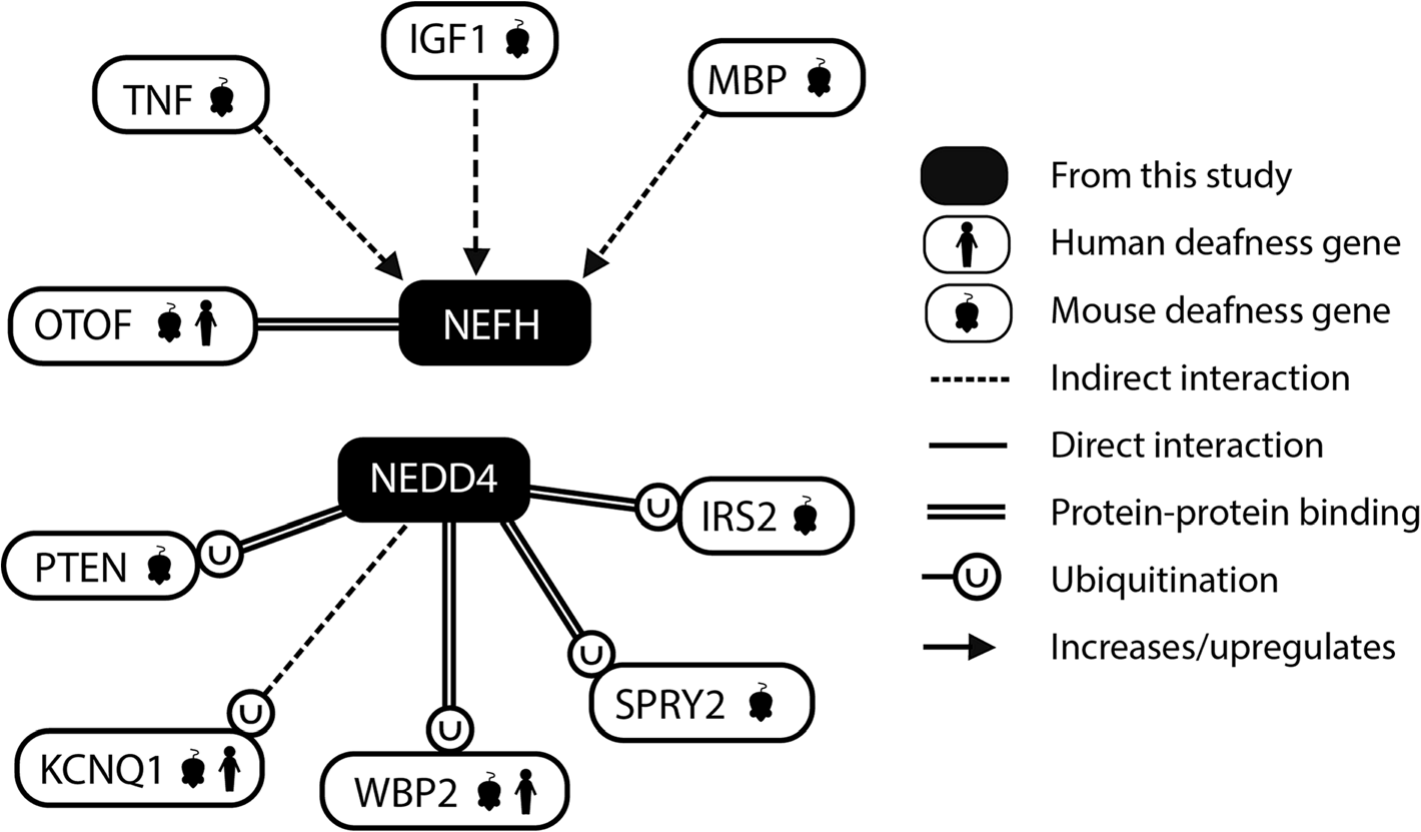
Network analyses of *NEFH* and *NEDD4. NEFH* has been linked to *TNF*, *IGF1* and *MBP* [58–60], which are all deafness genes in the mouse [58, 61, 62], and binds to OTOF [47], which is involved in deafness in mice and humans [48–50]. *NEDD4* binds to and ubiquitylates the products of the mouse deafness genes *PTEN*, *SPRY2* and *IRS2* [63–69], and two proteins implicated in deafness in both mice and humans, WBP2 and KCNQ1 [26, 40–44]

*NEDD4* is a particularly interesting novel candidate; it is known to be widely expressed in the cochlear duct [39], and the encoded protein is a ubiquitin ligase that binds to and ubiquitylates the products of several deafness genes, including *WBP2* [40] and *KCNQ1* [41], both of which are implicated in sensorineural deafness in humans and mice [26, 42–44] (Fig. 4). It is involved in AMPA receptor ubiquitination, playing a critical role in AMPA receptor trafficking in rat neurons [45]. The two variants are both missense, one resulting in a substitution of arginine for serine in exon 1, and the other is a substitution of valine for methionine towards the start of exon 1. Patients bearing these variants are all heterozygotes, and fall into the dominant and sensory subgroups.

NEFH, the neurofilament heavy chain, is expressed in the rat cochlear nucleus [46] and the spiral ganglion neurons [34]. It is known to bind to OTOF [47], which is involved in deafness in humans and mice [48–50], and its expression is affected by several other deafness genes (Fig. 4). We confirmed an 18 bp duplication (rs147489453) in eight people (homozygotes and heterozygotes, from the recessive, sensory and dominant subgroups), and a missense variant (rs59371099) in four people (heterozygotes and one homozygote, from the dominant and recessive subgroups) (Table 3). Four people carried both variants, three from the dominant subgroup and the one individual from the recessive subgroup who was heterozygous for both. The other two patients from the recessive subgroup with a variant in *NEFH* were homozygous for the 18 bp duplication (Table 3). Both variants are in the last exon of *NEFH* (exon 4), which has only one isoform. The 18 bp duplication is predicted to duplicate six amino acids in a low complexity region. The missense variant results in a substitution of lysine, which is positively charged, for glutamate, which is negatively charged, which may affect protein function, but without functional studies it is hard to predict what difference either variant would make to the function of the protein.

ZAN is a multiple-domain transmembrane protein found on the apical region of the sperm head [51], which functions to bind the sperm to the zona pellucida [52]. The MAM domains, D domains, EGF-like domains and mucin-like domains it contains all play a role in adhesion in other proteins, typically in cell-cell or cell-extracellular matrix binding [51]. Although ZAN is thought to be testis-specific, it may also be involved in cell-cell adhesion in the organ of Corti. Variants in *ZAN* were found in patients from each subgroup, all in the heterozygote state except for three dominant patients who were homozygous for a 35 bp deletion (g.7:100385563_100385597del).

DNAH2 is an axonemal dynein heavy chain, about which very little is known. Six missense variants, one intronic splice site SNV and one single base pair insertion were predicted in seven patients (MAF < 0.5%), and six further missense variants with a MAF < 10% were predicted in 5 patients, of which we have confirmed one missense variant present in the heterozygous state in two patients from the dominant and recessive subgroups.

### Expression and mouse mutations of novel candidate deafness genes

We examined the expression of our novel candidate deafness genes using the gEAR portal [53], which displays data from the mouse organ of Corti at postnatal day (P)0 [54] – P7 [55], and found that both *Nedd4* and *Zan* were detected during this period. *Nedd4* is expressed at reasonably high levels in sensory cells, and only slightly lower levels in non-sensory cells, in accordance with previous publications [39]. *Zan* has very little expression in the hair cells but is strongly expressed in the supporting cells at P0 [54]. *Nefh* is expressed in the neurons under the inner hair cells [34], and is commonly used as a neuronal marker [26]. *Dnah2* is expressed in the cochlear duct at P0, but not as strongly as *Zan* or *Nedd4*.

There are several mouse lines bearing mutations in *Nedd4*, *Zan,* and *Nefh*, but the only mouse line with a variant in *Dnah2* has a large inversion on chromosome 11 covering 1898 genes. No hearing phenotype has been reported for any of the mutant alleles of these genes (as recorded in the Mouse Genome Informatics database [56]), but these mice may not have been tested for auditory function.

## Conclusions

Our analysis has identified several candidate variants and genes for involvement in adult-onset progressive hearing loss, in particular variants in *NEFH* and *NEDD4*. It is perhaps surprising that our relatively small sample size of thirty individuals was able to identify good candidate variants and genes. By targeting our approach to specific phenotypic subtypes within large, well-characterised patient cohorts based on audiogram shape, family history and similar age of onset, we have sought to increase power to detect causal genetic variants. In addition, by cross-referencing our data in the filtering pipeline with data from the mouse we were able to prioritise the strongest candidate variants.

The two strongest candidates from our analysis, the variants in *NEDD4* and *NEFH*, should be followed up in larger cohorts and by functional studies to confirm whether they are causal mutations.

Our study also highlights the potential pitfalls of using targeted sequencing to diagnose the cause of adult-onset hearing loss in a typical clinical scenario, where relatives are not available for segregation analysis especially on a gene by gene basis. Of the 30 individuals WES was only able to identify the likely causal mutation in one individual with five WFS1 variants. For the remaining patients the variants identified are of uncertain pathogenicity without further validation. Interpretation of these variants in single individuals is extremely challenging given that even when we limited our analysis to dominantly-inherited human deafness genes, 2334 of the 2504 individuals in the 1000 Genomes Project data carried at least one predicted pathogenic variant in a known dominant human deafness gene. These findings reveal the need for allele frequency databases from carefully-selected controls with good hearing for their age rather than the existing unselected general population controls in studies of highly-prevalent disorders such as hearing loss. Here, we have explored the use of WES in undiagnosed individuals, based on our results it would be interesting to pursue a similar study in individuals who have received a genetic diagnosis to ascertain the number of other predicted pathogenic variants that are present in deafness genes. Furthermore, our study demonstrates that confirmation of candidate variants by Sanger sequencing is always a necessary step. Our findings further suggest that for diagnostics in a clinical situation, novel candidate variants identified by sequencing should be investigated by family analysis if possible, to look for segregation. Without these additional steps, our data suggest that it is not possible to determine with confidence the causative mutation responsible for a patient’s hearing impairment from exome sequence alone. Even with these secondary steps great caution should be exercised in interpreting predicted disease-causing variants, given our findings of the incidence of such variants in every genome.

## Additional files

Additional file 1: **Figure S1.** showing the audiograms of each participant in the recessive patient group. (PDF 486 kb)

Additional file 2: **Figure S2.** showing the audiograms of each participant in the dominant patient group. (PDF 485 kb)

Additional file 3: **Figure S3.** showing the audiograms of each participant in the metabolic and sensory patient groups. (PDF 428 kb)

Additional file 4: **Table S1.** which lists the software prediction tools used to call and annotate variants, with relevant references. (XLS 30 kb)

Additional file 5: **Figure S4.** and legend detailing counts at each filtering step for common variant analysis. (PDF 259 kb)

Additional file 6: **Table S2.** detailing primers used for confirming selected variants. (DOCX 20 kb)

Additional file 7: **Table S3.** listing genes reported to underlie deafness in humans and/or mice. (XLS 45 kb)

Additional file 8: **Table S4.** and lists candidates for exclusion - genes with predicted pathogenic variants in many exome projects or across multiple families exhibiting a variety of phenotypes. This list consists of the genes in Supplementary Tables S1 and S2 from [29]. (XLS 46 kb)

Additional file 9: **Table S5.** and lists human retinal disease genes, from RetNet [30]. (XLS 45 kb)

Additional file 10: **Table S6.** listing individuals homozygous for very rare variants in any gene. (DOCX 15 kb)

Additional file 11: **Table S7.** listing individuals with more than one very rare mutation in the same gene. (DOCX 20 kb)

Additional file 12: **Table S8.** giving details of the single very rare variants in known deafness genes found in multiple samples. (DOCX 16 kb)

Additional file 13: **Table S9.** giving details of genes with identical variants found in more than one person. (DOCX 16 kb)

Additional file 14: **Table S10.** detailing very rare mutations in GWAS candidates. (DOCX 20 kb)

Additional file 15: **Table S11.** which contains further details of the validated variants listed in Table 3. (XLS 32 kb)

Additional file 16: **Table S12.** giving details of very rare mutations identified in GPR98. (DOCX 19 kb)

## Abbreviations

ARHL: Age-related hearing loss
EGA: European Genome-Phenome Archive
GWAS: Genome-wide association studies
MAF: Minor allele frequency
MUSC: Medical University of South Carolina
WES: Whole-exome sequencing
