# Supplementary material for: Whole exome sequencing in adult-onset hearing loss reveals a high load of predicted pathogenic variants in known deafness-associated genes and identifies new candidate genes
Source: BMC Med Genomics. 2018 Sep 4;11:77. doi: 10.1186/s12920-018-0395-1 (PMC6123954; doi:10.1186/s12920-018-0395-1)
Supplement: Supplementary file 1 — Figure S1. showing the audiograms of each participant in the recessive patient group. (PDF 486 kb) [file 12920_2018_395_MOESM1_ESM.pdf]

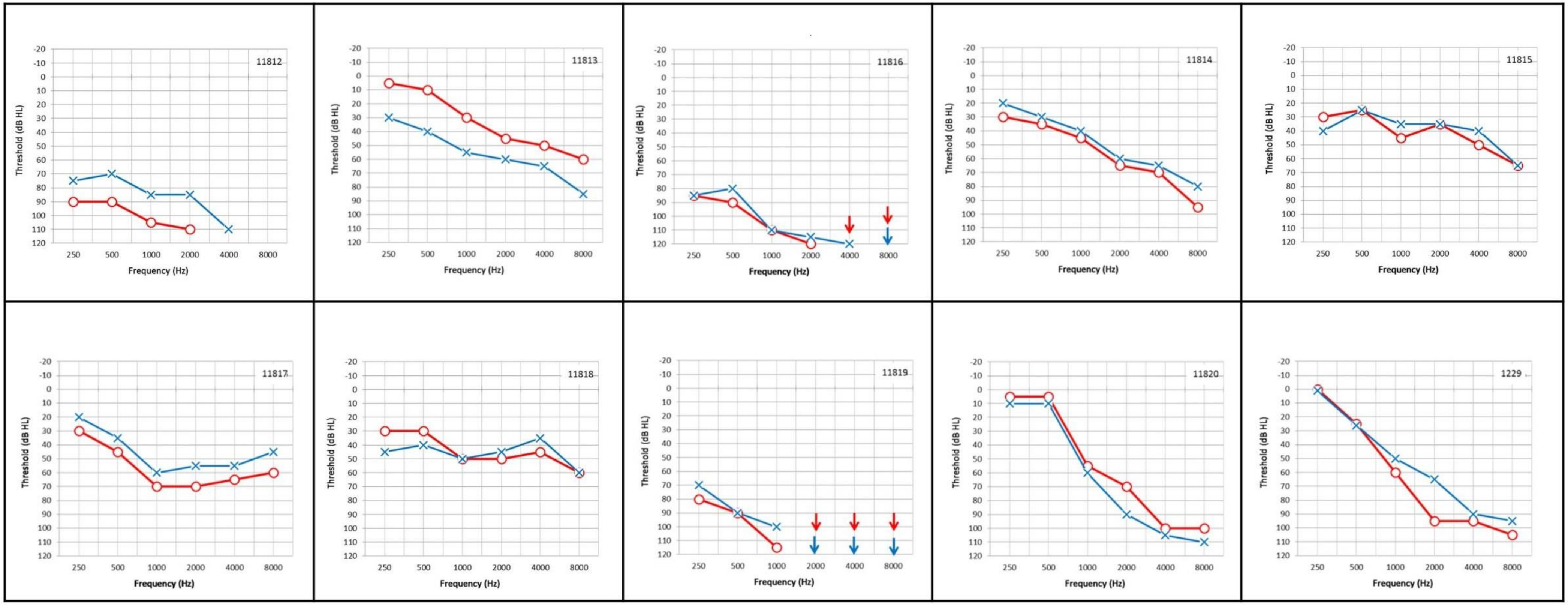

Figure S1. Description of data: Audiograms of the 10 patients in the *Recessive* patient group. Blue crosses and red circles show the thresholds of the left and right ear, respectively. Lack of any detectable response at a particular frequency is designated by a downward arrow.
