# Supplementary material for: Whole exome sequencing in adult-onset hearing loss reveals a high load of predicted pathogenic variants in known deafness-associated genes and identifies new candidate genes
Source: BMC Med Genomics. 2018 Sep 4;11:77. doi: 10.1186/s12920-018-0395-1 (PMC6123954; doi:10.1186/s12920-018-0395-1)
Supplement: Supplementary file 3 — Figure S3. showing the audiograms of each participant in the metabolic and sensory patient groups. (PDF 428 kb) [file 12920_2018_395_MOESM3_ESM.pdf]

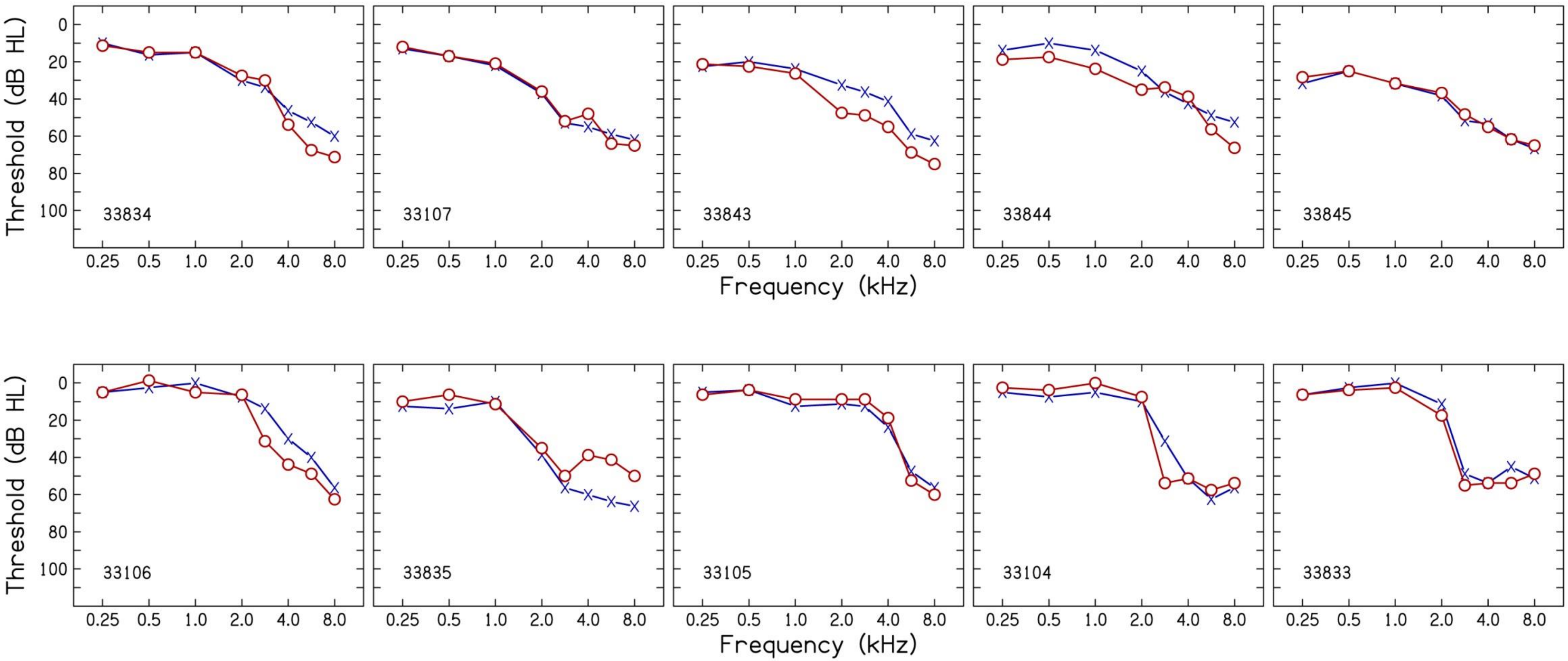

Figure S3. Description of data: Audiograms of the 10 patients in the *Metabolic* (top) and *Sensory* (bottom) groups. Blue crosses and red circles show the thresholds of the left and right ear, respectively.
