# Supplementary material for: Whole exome sequencing in adult-onset hearing loss reveals a high load of predicted pathogenic variants in known deafness-associated genes and identifies new candidate genes
Source: BMC Med Genomics. 2018 Sep 4;11:77. doi: 10.1186/s12920-018-0395-1 (PMC6123954; doi:10.1186/s12920-018-0395-1)
Supplement: Supplementary file 5 — Figure S4. and legend detailing counts at each filtering step for common variant analysis. (PDF 259 kb) [file 12920_2018_395_MOESM5_ESM.pdf]

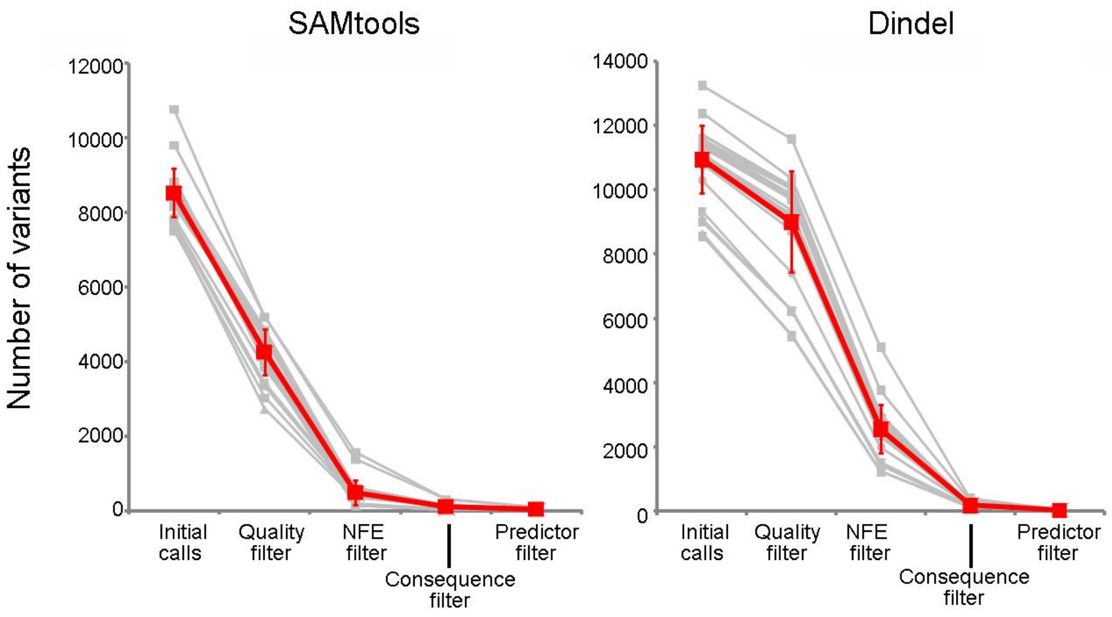

Figure S4. Counts of variants throughout filtering. Numbers of variants called by SAMtools (left) and Dindel (right) at each filtering step for common variant analysis. Individual sample counts are shown in grey, with the mean and standard deviation plotted in red on top.
