# Supplementary material for: Whole exome sequencing in adult-onset hearing loss reveals a high load of predicted pathogenic variants in known deafness-associated genes and identifies new candidate genes
Source: BMC Med Genomics. 2018 Sep 4;11:77. doi: 10.1186/s12920-018-0395-1 (PMC6123954; doi:10.1186/s12920-018-0395-1)
Supplement: Supplementary file 6 — Table S2. detailing primers used for confirming selected variants. (DOCX 20 kb) [file 12920_2018_395_MOESM6_ESM.docx]

Table S2: Primers used for confirming selected variants

| **Gene** | **Location** | **Forward primer** | **Reverse primer** |
| --- | --- | --- | --- |
| *ADC* | 1:33583653-33583679 | GGTTGATGGCTGTGATTGCG | AGTTTCCCTCGTTTCTGCCC |
| *PAX2* | 10:102587323-102587333 | AACGCAACTATTCTCCGGGG | GGGGTGATGTGAAGGGTTGC |
| *NEDD4* | 15:56208463-56208933 | TTGTTGGAGAAGTCGGTCGG | ACTTTGCAGCCAGAAGAAGC |
| *MON1B* | 16:77228709-77228721 | CACACTGGACCGACTTCTGG | ATCCCATCTTCAACCAGGCG |
| *DNAH2* | 17:7736242-7736480 | TTCAGCTCGCCAAAACAACG | ATGCCGATGACAAAGGAGGC |
| *RALBP1* | 18:9512985 | GATCCCAGTGCTTTCAACCC | CATCAGGAGGCTCATGGAGG |
| *SIRPA* | 20:1895964-1895965 | CACTGCGACCTCTCTGATCC | AAACCAGAGGCAAAGGAGGC |
| *NEFH* | 22:29885016-29885564 | TGGAAGGTGAAGAGTGTCGG | GGCCTTCTCTGGGGACTTAG |
| *LRBA* | 4:151727540 | AGTTCTTGTCCATGGCTACCC | ACTGCTCTTAACTGGCCAGG |
| *LRBA* | 4:151837565 | AGAGGTCAATTATTCAGTGATTTACGC | TCTGCTCATTTTGTTGGAGGC |
| *RBPJ* | 4:26426018 | CCTTCTTTTGCTCCCTCCCC | TCATCCACTGCAGTAGGTGG |
| *ZAN* | 7:100346094 | CCATCCCTTCTGTGACTGGG | GGACTGATCTTCCCACTGCC |
| *ZAN* | 7:100353014-100353014 | GGGGTAGAGGAGAGACAGGG | GATGCCACTACTCTCCAGCC |
| *ZAN* | 7:100371417-100371479 | CAGGAGGAAGGCTCAGAACC | CCCTTCTCAGCAGTTGGAGG |
| *ZAN* | 7:100385563 | GATTACAGGCACGCACCACC | CCATCTTGTTGCGTCCTTCC |
| *ZAN* | 7:100389590 | TCACTGCAGTTTCCACCTCC | CACTCTTGGAAGGGCTCTGG |
| *TMEM8B* | 9:35841783 | TGCCCAGCTTCACTTACAGG | TTCAGGGAGCATGAAGGAGC |
| *GPR98* | 5:90106415-90106418 | TGAGCCTGTTCAGAATGGGG | GTTGGTTGGCTGCAGAATGG |
| *GPR98* | 5:89925039 | GGGACAGGAAAAACACTGGC | CTTTTGCTTGCAAGGGGTCC |
| *PFKM* | 12:48516464 | AAAGGGAGGGATCGTTGAGG | AGCAAAGCCCAAAATTCCCG |
| *WFS1* | 4:6296783 | GGTTTCCTCCACCTGAACCC | CTCTTTGTTAGGCGCGAACC |
| *WFS1* | 4:6302459-6303119 | CCACGTACCATCTTTCCCCC | GAGGAAGTAGCCGATGGAGG |
| *WFS1* | 4:6290798 | CCTGGCTTTCTATGGTCCCC | TACAAGCTGCTCAACCCTCC |
| *GRM7* | 3:7494306 | CTGGCTATCACGTGGTAGGC | CGGTAGTCAGCACAGAGATCC |
| *SIK3* | 11:116728913 | GCTGTCATGGCAACCTTTGG | CCCAACAGAGTTTGCACAGC |
