# Supplementary material for: Whole exome sequencing in adult-onset hearing loss reveals a high load of predicted pathogenic variants in known deafness-associated genes and identifies new candidate genes
Source: BMC Med Genomics. 2018 Sep 4;11:77. doi: 10.1186/s12920-018-0395-1 (PMC6123954; doi:10.1186/s12920-018-0395-1)
Supplement: Supplementary file 10 — Table S6. listing individuals homozygous for very rare variants in any gene. (DOCX 15 kb) [file 12920_2018_395_MOESM10_ESM.docx]

Table S6. Individuals homozygous for very rare variants in any gene.

| **Individuals** | **Genes** | |  |  |  |  |  |
| --- | --- | --- | --- | --- | --- | --- | --- |
| 3 | *SIRPA* | |  |  |  |  |  |
| 2 | *ZAN* |  |  |  |  |  |  |
| 1 | *AKR7A2* | *ATG2A* | *CBX2* | *CNGA2* | *COPE* | *CSMD3* | *INPP5B* |
|  | *LRDD* | *MID1IP1* | *MPZL3* | *NAP1L3* | *NSDHL* | *OR4C11* | *HLA-DPB1* |
|  | *CXorf64* | *DDX26B* | *DUSP13* | *FAAH2* | *FBXO38* | *GABRQ* | *GPR64* |
|  | *OR6C4* | *PDK3* | *SVEP1* | *PGK1* | *PLIN4* | *SACS* | *SRA1* |

Candidates for exclusion are underlined.
