# Supplementary material for: Whole exome sequencing in adult-onset hearing loss reveals a high load of predicted pathogenic variants in known deafness-associated genes and identifies new candidate genes
Source: BMC Med Genomics. 2018 Sep 4;11:77. doi: 10.1186/s12920-018-0395-1 (PMC6123954; doi:10.1186/s12920-018-0395-1)
Supplement: Supplementary file 11 — Table S7. listing individuals with more than one very rare mutation in the same gene. (DOCX 20 kb) [file 12920_2018_395_MOESM11_ESM.docx]

Table S7. Individuals with more than one very rare mutation in the same gene.

| **Variants/gene** | **Gene** | **Sample ID** | **Group** |
| --- | --- | --- | --- |
| 5 | ***WFS1*** | 2590 | Dominant |
| 4 | *ACAD10* | 33844 | Metabolic |
| 4 | *DST* | 33844 | Metabolic |
| 4 | *FRAS1* | 33844 | Metabolic |
| 3 | *AHNAK* | 33844 | Metabolic |
| 3 | *CWF19L2* | 33844 | Metabolic |
| 3 | *DNAH17* | 33844 | Metabolic |
| 3 | *DNAH5* | 33844 | Metabolic |
| 3 | *FSIP2* | 33844 | Metabolic |
| 3 | *MYH15* | 33844 | Metabolic |
| 3 | *MYOM3* | 12229 | Recessive |
| 3 | *MYOM3* | 33844 | Metabolic |
| 3 | *OR51B4* | 33844 | Metabolic |
| 3 | *PLXNC1* | 12229 | Recessive |
| 2 | ***ACAN*** | 12229 | Recessive |
| 2 | ***ALMS1*** | 33844 | Metabolic |
| 2 | ***CACNB2*** | 33845 | Metabolic |
| 2 | ***CDH23*** | 2594 | Dominant |
| 2 | ***GPR98*** | 4363 | Dominant |
| 2 | ***GPR98*** | 33844 | Metabolic |
| 2 | ***GPR98*** | 11813 | Recessive |
| 2 | ***PCDH15*** | 12229 | Recessive |
| 2 | ***USH2A*** | 11814 | Recessive |
| 2 | 131 genes^a^ | - | - |

Known deafness genes are in bold, and candidates for exclusion are underlined. ^a^Genes not listed.
