# Supplementary material for: Whole exome sequencing in adult-onset hearing loss reveals a high load of predicted pathogenic variants in known deafness-associated genes and identifies new candidate genes
Source: BMC Med Genomics. 2018 Sep 4;11:77. doi: 10.1186/s12920-018-0395-1 (PMC6123954; doi:10.1186/s12920-018-0395-1)
Supplement: Supplementary file 12 — Table S8. giving details of the single very rare variants in known deafness genes found in multiple samples. (DOCX 16 kb) [file 12920_2018_395_MOESM12_ESM.docx]

Table S8. Details of the single very rare variants in known deafness genes found in multiple samples.

| **Genes** | **SNPID** | **Mutation** | **ExAC_ALL** | **Transcript and consequence** | **Number of individuals/ Group** |
| --- | --- | --- | --- | --- | --- |
| PAX2 | Insertion | 10:g.102587334dupC | - | ENST00000355243 (p.Gly381fs) | 3 recessive, 1 dominant |
| LRIG3 | Insertion | 12:g.59268352dupG | - | ENST00000320743 (p.Cys901fs) | 3 recessive |
| RBPJ | rs75014045 | 4:g.26426018C>G | 4.95E-05 | ENST00000342295 (p.Thr197Arg) | 3 dominant |
| ACAN | rs16942318 | 15:g.89382129C>A | 0.0178 | ENST00000352105 (p.Asp102Glu) | 1 recessive, 1 metabolic |
| DUOX2 | rs138774384 | 15:g.45392329C>T | 0.0004 | ENST00000389039 (p.Val1035Met) | 1 recessive, 1 dominant |
| GPR98 | rs61744480 | 5:g.89925039A>C | 0.0026 | ENST00000405460 (p.Ile508Leu) | 2 dominant |
| LAMA2 | rs73599293 | 6:g.129824406A>G | 0.0070 | ENST00000421865 (p.Asn2843Ser) | 1 recessive, 1 metabolic |
| LRIG1 | rs61751731 | 3:g.66436546C>T | 0.0139 | ENST00000273261 (p.Ala550Thr) | 1 recessive, 1 metabolic |
| MYO15A | rs200146361 | 17:g.18043906C>T | 0.0017 | ENST00000205890 (p.Arg1763Trp) | 1 recessive, 1 dominant |
| MYO6 | rs55662069 | 6:g.76596587C>T | 0.004 | ENST00000369985 (p.Thr845Ile) | 1 dominant, 1 metabolic |
| NTN1 | rs765779295 | 17:g.9066234A>C | 0.0019 | ENST00000173229 (p.Thr375Pro) | 1 sensory, 1 metabolic |
| PAX2 | Insertion | 10:g.102587333dupG | - | ENST00000355243 (p.Arg380fs) | 2 dominant |
| SLC9A3R1 | rs41282065 | 17:g.72758167G>A | 0.0019 | ENST00000262613 (p.Arg153Gln) | 2 dominant |

Details of the variants in known deafness genes. All insertions were predicted to cause a frame shift, and the other variants were nonsynonymous coding variants. ExAC_ALL indicates the Minor Allele Frequency of the variant in the Exome Aggregation Consortium data; - indicates variant is absent.
