# Supplementary material for: Whole exome sequencing in adult-onset hearing loss reveals a high load of predicted pathogenic variants in known deafness-associated genes and identifies new candidate genes
Source: BMC Med Genomics. 2018 Sep 4;11:77. doi: 10.1186/s12920-018-0395-1 (PMC6123954; doi:10.1186/s12920-018-0395-1)
Supplement: Supplementary file 13 — Table S9. giving details of genes with identical variants found in more than one person. (DOCX 16 kb) [file 12920_2018_395_MOESM13_ESM.docx]

Table S9. Genes with identical variants found in more than one person

| **Number of individuals** | **Genes** |  |  |  |  |  |
| --- | --- | --- | --- | --- | --- | --- |
| 11 | *MON1B* |  |  |  |  |  |
| 8 | *NEFH* |  |  |  |  |  |
| 6 | *UBE2O* | *ZMIZ2* | *PTGER4* |  |  |  |
| 4 | ***PAX2*** | *C12orf51* | *THRA* | *CHD3* | *LRBA* | *MSI1* |
|  | *ADC* | *PLXNC1* | *SIRPA* |  |  |  |
| 3 | ***LRIG3*** | ***RBPJ*** | *AOC2* | *AQP7* | *DAZAP1* | *EIF4G3* |
|  | *GPR124* | *LIMD1* | *LRBA* | *AMPD2* | *KRT79* | *KRT76* |
|  | *MYLK3* | *NNT* | *NTN3* | *PGM5* | *ZAN* | *TSR1* |
|  | *TSPYL5* |  |  |  |  |  |
| 2 | ***LRIG1*** | ***ACAN*** | ***MYO15A*** | ***MYO6*** | ***PAX2*** | ***LAMA2*** |
|  | ***NTN1*** | ***SLC9A3R1*** | ***DUOX2*** | ***GPR98*** | *+187^a^* |  |

This table shows the shared variants in all genes, with known deafness genes in bold and candidates for exclusion underlined. ^a^Genes not listed.
