# Supplementary material for: Whole exome sequencing in adult-onset hearing loss reveals a high load of predicted pathogenic variants in known deafness-associated genes and identifies new candidate genes
Source: BMC Med Genomics. 2018 Sep 4;11:77. doi: 10.1186/s12920-018-0395-1 (PMC6123954; doi:10.1186/s12920-018-0395-1)
Supplement: Supplementary file 14 — Table S10. detailing very rare mutations in GWAS candidates. (DOCX 20 kb) [file 12920_2018_395_MOESM14_ESM.docx]

Table S10. Very rare mutations in GWAS candidates.

|  | *GRM7* | *SIK3* | *ACAN* | *ACAN* | *ACAN* |
| --- | --- | --- | --- | --- | --- |
| **Group** | Metabolic | Metabolic | Metabolic and recessive | Recessive | Sensory |
| **Sample ID** | 33834 | 33844 | 33844 and 12229 | 12229 | 33104 |
| **Chr** | 3 | 11 | 15 | 15 | 15 |
| **Position** | 7494306 | 116728913 | 89382129 | 89392939 | 89401184 |
| **VEP Consequence** | FRAMESHIFT_CODING | NON_SYNONYMOUS_CODING | NON_SYNONYMOUS_CODING | NON_SYNONYMOUS_CODING | NON_SYNONYMOUS_CODING |
| **Transcript ID** | ENST00000357716 | ENST00000292055 | ENST00000439576 | ENST00000439576 | ENST00000439576 |
| **Gene Consequence** | c.1187delT | c.C2950T | c.C306A | c.G2003A | c.T5368A |
| **Protein Consequence** | p.I396fs | p.R984C | p.D102E | p.R668Q | p.S1790T |
| **ExAC_ALL** | - | 0.0078 | 0.0178 | 0.0035 | 0.0018 |
| **ExAC_NFE** | - | 0.0002 | 0.0009 | 0.0001 | 0.0031 |
| **SNPID** | - | rs61738656 | rs16942318 | rs77572130 | rs201149160 |

*VEP* is Ensembl Variant Effect Predictor. ExAC_ALL shows the Minor Allele Frequency (MAF) of the variant in the Exome Aggregation Consortium data, all samples; ExAC_NFE is the MAF of the variant in Non-Finnish European samples only.
