# Supplementary material for: Whole exome sequencing in adult-onset hearing loss reveals a high load of predicted pathogenic variants in known deafness-associated genes and identifies new candidate genes
Source: BMC Med Genomics. 2018 Sep 4;11:77. doi: 10.1186/s12920-018-0395-1 (PMC6123954; doi:10.1186/s12920-018-0395-1)
Supplement: Supplementary file 16 — Table S12. giving details of very rare mutations identified in GPR98. (DOCX 19 kb) [file 12920_2018_395_MOESM16_ESM.docx]

Table S12. Details of very rare mutations identified in *GPR98*.

| **SNPID** | **Gene Consequence** | **Protein Consequence** | **ExAC_ALL** | **ExAC_NFE** |
| --- | --- | --- | --- | --- |
| rs145556097 | c.G3151T | (p.Asp1051Tyr) | 0.0022 | 0.0031 |
| rs61744480^a^ | c.A1522C | (p.Ile508Leu) | 0.0026 | 0.0035 |
| rs749622020 | c.C18629T | (p.Pro6210Leu) | 1.08E-05 | 1.94E-05 |
| rs765063974 | c.T15338G | (p.Leu5113Arg) | 8.31E-06 | 1.50E-05 |
| rs752508814 | c.A15341G | (p.Asn5114Ser) | 8.31E-06 | 1.50E-05 |
| rs773357747 | c.T17857-6C | splice region | 0.0002 | 0.0005 |
| rs199839743 | c.C12269A | (p.Thr4090Asn) | 0.0025 | 0.004 |
| rs61745498 | c.C581A | (p.Pro194His) | 0.0065 | 0.0001 |
| rs73181648 | c.G3956A | (p.Arg1319Gln) | 0.0011 | - |

^a^The missense variant found in 2 individuals. Gene and protein consequences refer to transcript ENST00000405460.
